# Heavy Metal Induced Oxidative Stress Mitigation and ROS Scavenging in Plants

**DOI:** 10.3390/plants12163003

**Published:** 2023-08-20

**Authors:** Sheikh Mansoor, Asif Ali, Navneet Kour, Julia Bornhorst, Khadiga AlHarbi, Jörg Rinklebe, Diaa Abd El Moneim, Parvaiz Ahmad, Yong Suk Chung

**Affiliations:** 1 Department of Plant Resources and Environment, Jeju National University, Jeju 63243, Republic of Korea; mansoorshafi21@gmail.com; 2 Biomedical Sciences Research Institute, School of Biomedical Sciences, Ulster University, Coleraine BT52 1SA, Northern Ireland, UK; asifalee1433@gmail.com; 3 Division of Biochemistry, Sher-e-Kashmir University of Agricultural Sciences and Technology of Jammu, Jammu 180009, India; 4 Food Chemistry, Faculty of Mathematics and Natural Sciences, University of Wuppertal, 20, 42119 Wuppertal, Germany; bornhorst@uni-wuppertal.de; 5 Trace Age-DFG Research Unit on Interactions of Essential Trace Elements in Healthy and Diseased Elderly (FOR 2558), Berlin-Potsdam-Jena-Wuppertal, 14558 Nuthetal, Germany; 6 Department of Biology, College of Science, Princess Nourah bint Abdulrahman University, P.O. Box 84428, Riyadh 11671, Saudi Arabia; kralharbi@pnu.edu.sa; 7 Laboratory of Soil and Groundwater Management, Institute of Foundation Engineering, Water and Waste Management, School of Architecture and Civil Engineering, University of Wuppertal, Pauluskirchstraße 7, 42285 Wuppertal, Germany; rinklebe@unwppertal.de; 8 Department of Plant Production (Genetic Branch), Faculty of Environmental Agricultural Sciences, Arish University, El-Arish 45511, Egypt; dabdelmoniem@aru.edu.eg; 9 Department of Botany, Government Degree College, Pulwama 192301, Jammu and Kashmir, India

**Keywords:** heavy metals, toxicity, stress, signaling, ROS, mitochondrial dysfunction, cell death

## Abstract

Although trace elements are essential for life, environmental contamination due to metal accumulation and overuse in various sectors, such as healthcare, agriculture, industry, and cosmetics, poses significant health concerns. Exposure of plants to heavy metals leads to the overproduction of reactive oxygen species (ROS) due to their ability to change mitochondrial membrane permeability and restrict the action of ROS clearance enzymes in the cellular antioxidant system. The interaction of ROS with cellular membranes, heavy-metal-induced interactions directly or indirectly with different macromolecules, and signaling pathways leads to the accumulation of environmental pollutants and oxidative stress in exposed organisms. The heavy metal–ROS–cell signaling axis affects various pathological processes such as ATP depletion, excess ROS production, mitochondrial respiratory chain damage, decoupling of oxidative phosphorylation, and mitochondrial death. This review focuses on discussing the toxic effects of different heavy metals on plants, with particular emphasis on oxidative stress, its consequences, and mitigation strategies.

## 1. Introduction

Molecules that possess at least one atom of oxygen and have unpaired electrons are referred to as reactive oxygen species (ROS). These contain singlet hydroxyl, oxygen, and hydroperoxyl radicals [1,2]. ROS are formed due to the incomplete decomposition of molecular oxygen like hydroxyl radicals (OH), hydrogen peroxide (H_2_O_2_), superoxide radical anion (O^2−^), and ozone (O_3_) [3]. ROS, being essential for various signaling pathways, are generated within cells produced via a wide range of physiological and biochemical processes. In aerobic life, reactive active species tolerate an essential chemical entity. In plants, any variations in enzymes and cellular structure, nucleic acid, or proteins, increase the production of ROS. In plants, ROS scavenging pathways include enzymatic and non-enzymatic action regulating the production of ROS [4,5,6]. Therefore, under unfavorable conditions, the higher plants are induced to produce ROS to promote an extensive range of physiological variations. Production of ROS under the impact of drought, salinity, heavy metal toxicity, etc., leads among others, to the degradation of antioxidants, ultimately induces the gene expression of antioxidative response genes, and may damage macromolecules as lipids (lipid peroxidation) or the DNA [7,8,9,10,11].

Anthropogenic activities like urbanization and industrialization are accompanied by the release of heavy metal pollutants into the environment, which in turn disturbs plant development and physiology by phytotoxicity [9]. Additionally, secondary metabolites counterattack ROS to maintain balance. For example, vitamins, terpenes, and polyphenols are chief secondary metabolites to counterattack ROS and inhibit oxidative strain in plants [7,10]. Presently the heavy metal toxicity to plant physio-chemical activities are highly concerning due to its toxicity accumulation in the food chain [12,13]. To reduce oxidative stress due to heavy metals, plants have evolved several mechanisms such as increased root extraction of metals, prohibiting metal entrance into the plant, preventive toxic metal accretion, chelation by organic compounds sequestration in vacuoles, and metal binding in the cell wall to stop the entry [14] and ROS are converted to lesser toxic compounds [15,16].

Heavy metals naturally exist in the Earth’s crust with varying concentrations and are not easily broken down into less toxic compounds through metabolic processes. When present in the soil, they persist for extended periods, causing harmful impacts on both the environment and human health [17,18]. In spite of these external effects, heavy metals alter many physiological processes, nutritional status of minerals, rate of photosynthesis and respiration, enzymatic processes, and many biochemical processes. However, on exposure of plants to toxic concentrations of heavy metals, more production of reaction oxygen species (ROS) is considered the most prompt effect so far. Heavy metals interact with various components in the electron transport chain thereby altering its activity and leading to ROS generation mainly in chloroplast and mitochondria [19,20,21]. With the rise in ROS level, the membrane potential gets imbalanced further inducing leakage of ion channels, lipid peroxidation, and destruction of macromolecules. Moreover, the toxicity of heavy metals liberating ROS on subcellular organelles may alter depending upon certain factors like duration of stress, developmental stage, dose/concentration of particular heavy metal, and plant organ involved [22,23].

Apart from these unfavorable conditions, ROS are also produced in plants at a certain basal rate which does not account for any toxic effect because of scavenging via different antioxidant mechanisms. Due to the immobile nature of plants, all the available resources including environmental contaminants mainly heavy metals are taken up. Further, they are distributed in different tissues and hence contribute to negligible toxicity inside the plant [24,25]. Due to the consumption of plants contaminated with heavy metals, animals and even humans also accumulate them in different tissues at high toxic levels. Hence, understanding the mechanism or the pathways that contribute to the limited uptake of heavy metals in plants is of paramount concern. In recent years, manifold progress has been achieved regarding signaling pathways evolved in plants and their physiological response to heavy metal stress. This review emphasizes the significant progress made in understanding the signaling pathways and physiological responses of plants to heavy metal stress. The key events discussed include the activation of antioxidant defense systems, the production of non-enzymatic antioxidants, metal sequestration, and the regulation of metal uptake and transport. These strategies collectively contribute to the plant’s ability to mitigate heavy-metal-induced oxidative stress and protect its cellular structures from damage.

## 2. Mechanism of ROS Production

In living cells, metabolic processes can give rise to ROS as by-products, and their enhanced level leads to a state of oxidative burst. In plants, recent studies have demonstrated an interest in the dual function of ROS [19,26,27,28]. In addition to its detrimental effects, ROS plays a significant role in signaling molecules to regulate the process of cell growth and development, apoptosis as well as stimulation of systemic response under biotic and abiotic stress. As a result, enhanced level of ROS with disturbance in cellular redox potential leads to manifold oxidative stress responses in plants. The stress responses are modulated by the type of heavy metal, its uptake, and its concentration [29,30,31]. On the perception of stress signal, plant adapts various mechanisms to adapt and survive under severely adverse conditions.

The generation and accumulation of ROS, hydroxyl radical, singlet oxygen, superoxide anion, and hydrogen peroxide act as a secondary industry in signaling pathways by regulating the process of stomatal closure, apoptosis, and senescence thereby affecting the plant growth and development [32,33,34]. On exposure to heavy metal stress, an alarming level of oxidative burst due to elevated ROS generation arises. This will further lead to the activation of transcription factors, and altered gene expression related to the defense mechanism. The condition of oxidative burst causes severe damage to macromolecules like DNA and proteins due to which the normal functioning of plants, as well as animals, gets disturbed [35,36,37]. The concentration of ROS is the key event in signaling, the higher conc. is mainly responsible for programmed cell death whereas it acts as a signal in mediating the stress response in lower/moderate concentrations [8,38,39].

The presence of oxidoreductases, specifically NADPH oxidase at the plasma membrane, is the chief source of ROS generation in plants. It accepts the electron from NADPH and stimulates the production of superoxide radicals in the apoplastic region that is further responsible for diverse biological processes as well as signal transduction. The NADPH oxidases are categorized as respiratory burst oxidase homologs (Rboh)—the fundamental enzymes required for the transfer of electrons from NADPH to O_2_ which thereby stimulate the generation of superoxide radical followed by dismutation into H_2_O_2_ [40]. As CDPKs lie upstream of NADPH oxidases, hence cause their phosphorylation on sensing heavy metal stress. This leads to the subsequent elevation of apoplastic superoxide and activation of MAPK cascade for downstream signaling [40,41,42,43]. A study proposed by [44] noted the NADPH oxidase activity only after 6 and 24 h of cadmium stress in plants. Whereas the activation of the MAPK cascade gene was visualized after a period of 3 h.

## 3. Antioxidative Defense System in Plant Cell Components

**Figure 1:**
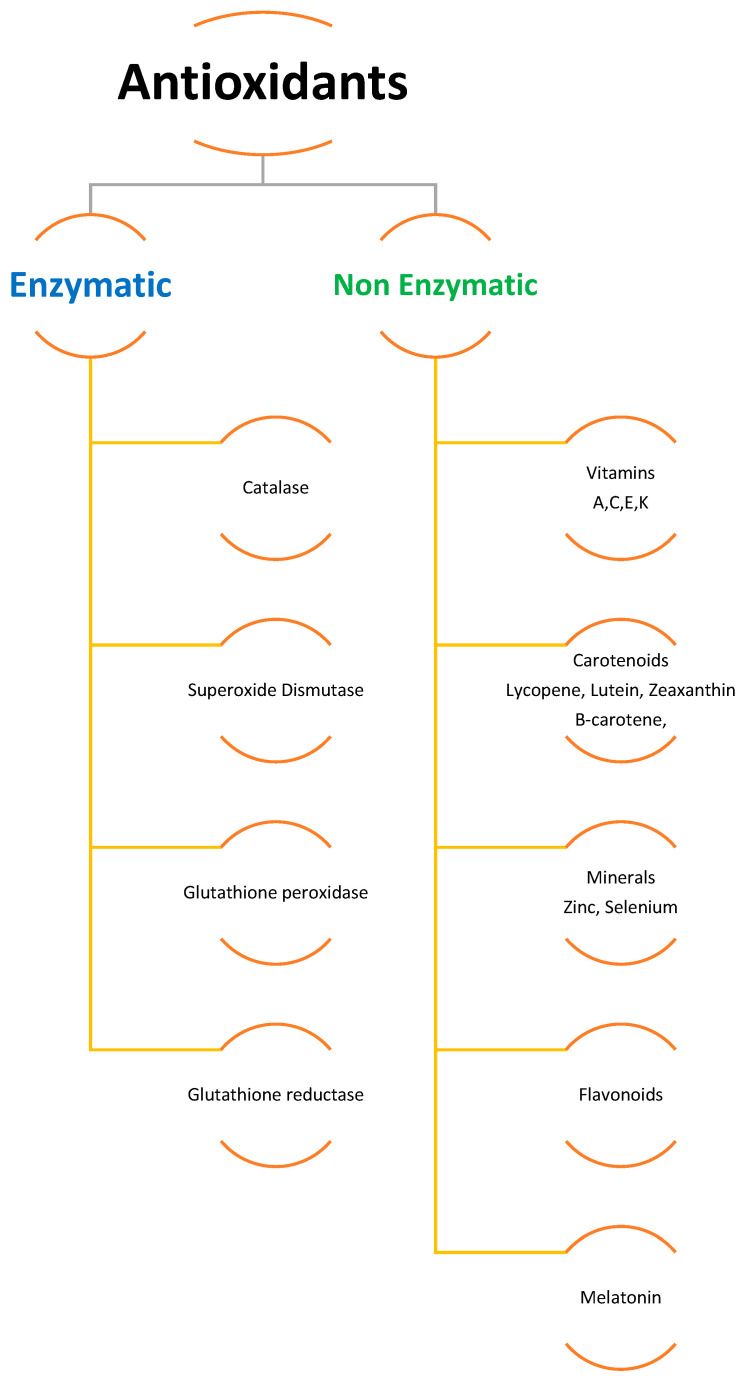
Enzymatic and non-enzymatic antioxidants.

To scavenge ROS, plants have a sophisticated antioxidative defense system that includes both non-enzymatic and enzymatic components [6,31,34,36]. Different organelles in plant cells, such as chloroplasts, mitochondria, and peroxisomes, have distinct ROS-generating and scavenging systems. Different cellular compartments’ ROS scavenging processes are coordinated. Potentially harmful oxygen metabolites are produced at a low level under normal settings, and there is an optimal balance between ROS production and quenching [36]. Several negative environmental conditions can disrupt the equilibrium between ROS production and quenching, resulting in fast increases in intracellular ROS levels. ROS are created in larger quantities in cellular compartments in the presence of transition heavy metals within the cell and many other environmental stress conditions, causing oxidative stress in plants and causing damage to cell protein, lipids, and nucleic acid [7,13,36,38,45,46,47]. Cells employ enzymatic (e.g., CAT, GSH, SOD, APX, GPX) and non-enzymatic antioxidants (e.g., ascorbate, carotenoids, flavonoids, phenolics) to counter ROS damage (Figure 1). It was also discovered that antioxidants such as superoxide dismutase (SOD) and glutathione reductase (GR), ascorbate peroxidase (APX), and peroxidase (POD) are produced inside plants as a result of metal build-up. Additionally, research has shown that plants create more enzymatic and non-enzymatic antioxidants as a natural defense against exposure to increased concentrations of heavy metals and other abiotic stressors [31,34,48]. It was also observed that antioxidants such as superoxide dismutase and glutathione reductase, ascorbate peroxidase, and peroxidase are created within the plant as a result of metal build-up. Together, these systems protect cells from oxidative stress [49].

Non-enzymatic antioxidants work by altering cellular metabolic functions to interact with polyunsaturated lipid acyl groups, thus stabilizing membranes. This helps protect against ROS generated from photosynthesis and respiration, working in tandem with other antioxidants [50]. Among them, lipid peroxidation causes the most damage, potentially leading to biomembrane deterioration. Malondialdehyde, a byproduct of polyunsaturated fatty acid breakdown in membranes, is used as an indicator of oxidative stress. Notably, plants produce low-molecular-weight thiols during heavy metal stress, establishing a strong connection to toxic metals [49,50,51].

Ascorbic acid (AsA) is a potent non-enzymatic antioxidant that effectively counteracts the detrimental effects of reactive oxygen species (ROS) due to its stability and ability to donate electrons. It plays a significant role in various biological processes, including the synthesis of phytohormones and the regeneration of alpha tocopherol. Ascorbic acid achieves detoxification by neutralizing hydroxyl and superoxide radicals, as well as tocopherol radicals [33,50,52]. Glutathione (GSH) is vital in the AsA-GSH cycle, scavenging diverse free radicals and maintaining cellular redox balance [33,47,53]. Among low-molecular-weight thiols, glutathione and cysteine are particularly effective. Glutathione contributes significantly to the detoxification of metals like nickel and cadmium and serves as a substrate for phytochelatin production, peptides with metal-binding properties [54,55].

The biosynthesis of AsA primarily involves the l-galactose pathway, with the VITAMIN DEFECTIVE 2 (VTC2) gene encoding GDP-l-galactose phosphorylase as a key player [56]. Additionally, jasmonic acid stimulates AsA biosynthesis by inducing the expression of the VTC2 gene. In the pathway of AsA recycling, transcription levels of APX genes significantly influence ROS balance within plant cells [57]. These genes are categorized based on their subcellular location, with cytosolic (APX1, APX2, APX6), microsomal (APX3, APX4, APX5), and chloroplastic (sAPX, tAPX) isoforms actively participating in ROS homeostasis. Moreover, Vitamin E is a lipid-soluble antioxidant that protects cell membranes from oxidative damage by scavenging lipid peroxyl radicals [58]. Various phenolic compounds, such as flavonoids and polyphenols, are known for their antioxidant properties. Heavy metal stress caused an increase in the activity of phenylalanine ammonia-lyase pathway (PAL) and tyrosine ammonia-lyase (TAL) enzymes and an increase in the accumulation of phenolic compounds [59]. Melatonin (N-acetyl-5-methoxytryptamine), a biostimulant, plant growth regulator, and antioxidant, enhances plant resilience against heavy metal stress. It achieves this by improving redox balance, nutrient levels, osmotic equilibrium, and metabolic processes. When applied externally, melatonin counters heavy metal toxicity by boosting protective gene expression, leading to heightened antioxidant actions and metal-binding properties [60,61]. Melatonin (MT) serves as a versatile signaling molecule that shields plants from the adverse consequences of heavy metals (HMs) in the soil. In the soil–water matrix, plants often encounter and quickly absorb HMs [62]. Table 1 outlines the distinct mechanisms by which enzymatic and non-enzymatic antioxidants function.

## 4. Heavy Metal Stress Signaling Events in Plants

Plants in response to heavy metal stress, initiate a manifold of signaling events that are mainly comprised of recognizing the signal, activation of downstream components and thereby neutralizing the detrimental effects. These events eventually regulate the cellular response at physiological, biochemical, and molecular levels [29,36,52,63]. Heavy metal stress is known to regulate a complex of signaling cascades like calcium/calmodulin-dependent, ROS-mediated signaling, hormone signaling, and mitogen-activated protein kinases (MAPK) that further mediate the phosphorylation of certain genes [38]. Heavy metals enter the plant system mainly through roots via diffusion with the cell wall binding to heavy metal ions at the sites with negative charge including –COOH, -OH, and -SH. Altered cell wall constituents may lead to the destruction of the cell membrane. Moreover, the complex formed by the interaction of heavy metal and functional groups may cause the disruption of plasma membrane integrity [53,64,65,66]. On perceiving the signal, various defense responses are triggered downstream. In a proteomic study conducted by [46] T. Liu et al. (2014), cell walls of E. splendens under copper stress indicated 40% of cell wall proteins in abundance that are specifically involved in cell wall modulation, antioxidant defense, and many other metabolic processes. Whereas the remaining 60% of these differentially expressed proteins are inadequate and play a role in translation, cell signaling, and energy synthesis. Another group of G-proteins (Hsp70 and RAS) has demonstrated a significant role in signal transduction under copper stress. The overall study demonstrated the potential of cell walls in inducing heavy metal stress signaling responses downstream [2,67]. Furthermore, the role of cell walls in signaling is spotlighted with the findings of integral membrane proteins namely aquaporins and kinases. The aquaporin gating was detected immediately upon exposure to heavy metals in the case of onion epidermal cells regardless of the type of metal [68,69,70]. The findings also suggest that a high concentration of zinc downregulates specifically AQUA1 (mercury-sensitive aquaporin). As per the observations, zinc stress leads to the regulation of intracellular signaling, post-translational modifications with the relocalization of AQUA1 [71].

Another component is secretory vesicles present beneath the plasma membrane which also contributes to the disruption of the cell wall under heavy metal stress. Consequently, it causes cell growth inhibition; therefore, it is necessary to understand the vesicular system as well as how it affects cell wall signaling [72,73]. The interaction among intracellular trafficking proteins namely molecular motors and cytoskeletal elements, microtubules, actin filaments, and intermediate filaments is responsible for vesicle transport. A study based on vesicle trafficking in root hairs of *A. thaliana* demonstrated the prominent role of cytoplasmic calcium gradient with actin filaments [74]. Treating root hairs specifically with cadmium using FM4-64 dye as fluorescence labeling, a clear disruption of endocytosis and membrane recycling was determined in *A. thaliana*. To better understand the reason behind the disruption, the study was applying in vivo labeling using confocal microscopy. The results obtained demonstrated the altered longitudinal positioning of actin filaments which is modified to the transverse arrangement, thereby affecting the vesicular trafficking system. Furthermore, it was found that cadmium imitates the action of calcium by binding to gelsolin, an actin-modulating protein [75,76]. In the root hairs of *A. thaliana*, the disruption of calcium channels, depolymerization of actin filaments as well as diminished vesicle trafficking were the key findings [77,78]. In *Funaria hygrometrica*, the process of internalization of pectin in the apical tip under lead stress specifically has been evaluated, by targeting pectin epitope *JIM5-P* due to its high affinity for lead using FM4-64 dye and immunogold labeling. The overall study reported by [79] Krzesłowska, demonstrated that lead accumulation causes enhancement of vesicular trafficking with the internalization of *JIM5-P* in the plasma membrane as well as in different vesicles. This pectin-based signaling is regarded as a universal response in other plant species on exposure to lead stress [79,80,81].

**Figure 2:**
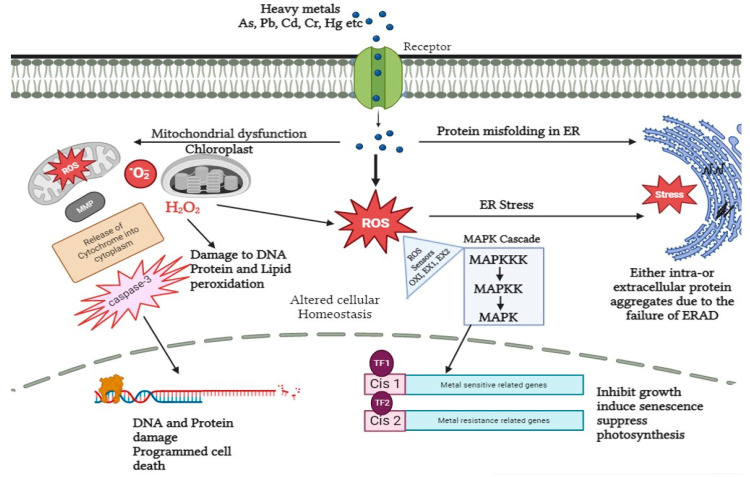
Signal transduction in response to heavy metal stress in plants. Heavy metals can affect ROS accumulation via dysfunction in the mitochondria and chloroplasts, which results in dysfunction in the degradation of ER-associated proteins and accumulation of aggregates. The accumulated ROS is detected by sensor (OX1, EX1, EX2) proteins and used as a signal to induce MAP Kinase cascade, which in turn causes the activation and inhibition of various downstream pathways.

After the root system, heavy metals enter the plasma membrane either by diffusion or by transporters and thereby downstream signal transduction occurs. Calcium sensors and potassium transporters are the known sensing receptors embedded in the plasma membrane of *A. thaliana*. Both are commonly known as transceptors [82,83]. In addition to this, *NRT1* and *SULTR1* are responsible for sensing nitrate and sulfur, respectively, in plants [84,85]. Apart from sensors and receptors, calcium-dependent protein kinases (CDPKs) also play a vital role in heavy metal signaling. On perceiving numerous stress responses, CDPKs transduce the signal to activate the downstream phosphorylation cascade and other proteins like NADPH oxidase, membrane channels, and transcription factors [86]. In leaves of Cucurbita pepo and seedlings of *Setaria italica* under nickel and chromium stress, respectively, overexpression of CDPKs gene expression has been detected. Moreover, CDPKs are essential components for stimulating the action of mitogen-activated protein kinases (MAPK) in the case of cadmium and copper stress by developing a close association between CDPKs and MAPKS, thereby providing a platform for subsequent signaling under heavy metal stress. MAPKs like CDPKs are localized at different cellular locations like cytosol, nucleus, microtubules, and plasma membrane. Herein the stress signal is directly transmitted to the nucleus. In *Nicotiana tabacum* under cadmium stress, upregulation of MAPK, as well as elevated tolerance level, has been detected [36,87,88,89,90]. Besides this, it also regulates the gene expression related to cell cycle events in *Oryza sativa*. Furthermore, in a study proposed by Xu et al. (2019) MAPK transcript was analyzed via real-time quantitative polymerase chain reaction (RT-PCR) in roots of *B. papyrifera* under cadmium stress. The results collected were with respect to time and clearly indicated the downregulation and upregulation of MAPK transcript at time periods of 3 h and 6 h, respectively [91,92]. This led the researchers to a clear conclusion about how MAPK’s recovery performs a significant task in downstream signaling events and also creates deleterious effects on plants on prolonged suppression of MAPK (Figure 2).

To scavenge ROS, plants have a sophisticated antioxidative defense system that includes both non-enzymatic and enzymatic components. Different organelles in plant cells, such as chloroplasts, mitochondria, and peroxisomes, have distinct ROS-generating and scavenging systems. Different cellular compartments’ ROS scavenging processes are coordinated [26,93,94]. Potentially harmful oxygen metabolites are produced at a low level under normal settings, and there is an optimal balance between ROS production and quenching [10,52,95].

## 5. Heavy Metal Mitigation Strategy

Heavy metal pollution including Cd, Cu, Zn, Ni, Co, Cr, Pb, and As has been detected in cultivated fields in many parts of the world. Long-term usage of phosphatic fertilizers, industrial wastewater sludge discharge, dust from anthropogenic sources, and inadequate irrigation practices in agricultural areas are all potential sources [96]. When plants are exposed to high quantities of heavy metals, all are potential sources of reactive oxygen species (ROS). Various metals either directly or indirectly generate ROS through Haber–Weiss reactions or overproduce ROS, causing oxidative stress in plants as an indirect result of heavy metal toxicity [6,97]. While Co, Cu, Fe, Mn, Mo, Ni, V, and Zn constitute merely a trace need for life, large concentrations of these metals can be harmful. Through absorption at the primary producer level and later consumption at the consumer level, metals are accumulated in the ecological food chain [31,72]. In aquatic environments, the plant body is exposed to these ions, and particles that are deposited on the foliar surfaces cause heavy metals to be directly integrated into the leaves [34,98].

Plants, on the other hand, have evolved a potentially useful strategy to mitigate environmental heavy metal toxicity. Low-molecular-weight thiols are produced by plants and have a great attraction to potentially deleterious metals. The two most significant low-molecular-weight biological thiols are cysteine and glutathione (GSH). GSH is a glutamate-cysteine-glycine tripeptide thiol that contains sulfur. The production of GSH is catalyzed by the ATP-dependent enzymes glutamylcysteine synthetase (GSH1) and glutathione synthetase (GSH2) [99,100]. GSH is essential for heavy metal detoxification, such as cadmium and nickel, and is a precursor to phytochelatin synthesis [21,101]. Phytochelatins are cysteine-rich polypeptides with a general structure of (-Glu-Cys) n Gly (n = 2−11) that bind heavy metals. PCs can be found in fungi and other species in addition to plants. The enzyme phytochelatin synthase catalyzes their synthesis (PCS). In the cytosol, PCs form complexes with harmful metal ions and then transport them into the vacuole [102]. As a result, these compounds protect plants from the harmful effects of heavy metals. Different types of environmental stresses, such as strong exposure, increasing or decreasing temperature, salinity, dehydration, nutritional deficiencies, and pathogen attack, cause the production of ROS in plants. Antioxidants, antioxidative enzymes, and other minute compounds have evolved in plants and other living beings to safely dissipate ROS [8]. Oxidative stress is caused by an imbalance between ROS generation and their removal by enzymatic and non-enzymatic processes. Photo oxidative damage to DNA, proteins, and lipids occurs as a result of increased net ROS generation, leading to cell death. ROS are also involved in pathogen defensive responses such as hypersensitive reaction and systemic acquired resistance, as well as stress hormone synthesis, desensitization, and programmed cell death [13,64,91,103,104,105,106,107,108].

## 6. Heavy Metal Remediation for Plant Growth Improvement

**Figure 3:**
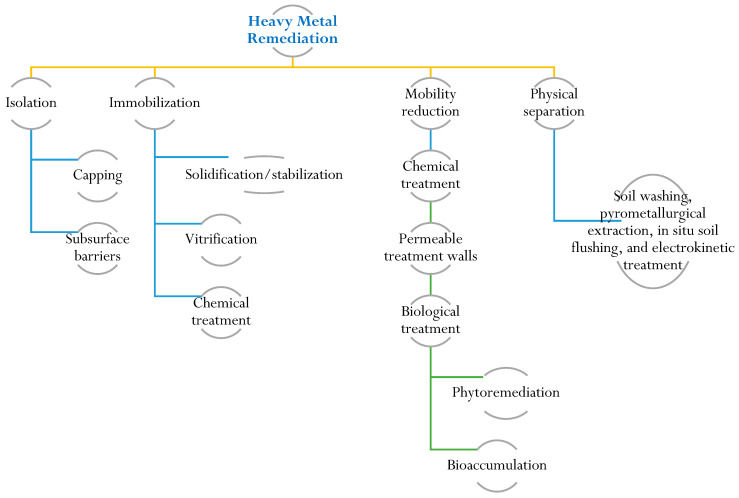
Illustration of remediation techniques for heavy metal contamination. Remediation techniques for heavy-metal-contaminated sites include physical, chemical, and biological methods.

To protect and restore soil ecosystems contaminated by heavy metals, it is crucial to understand their characteristics and remediation. Soil characterization provides insights into heavy metal speciation and bioavailability. Remediation efforts require knowledge of contamination sources, chemical properties, and associated environmental and health risks. Risk assessment is a valuable tool for the cost-effective management of contaminated sites, ensuring the preservation of public and ecosystem health [109]. Remediation techniques for heavy-metal-contaminated sites encompass various approaches, including physical, chemical, and biological methods. Physical methods involve actions like excavation and containment to physically remove or isolate contaminated soil. Chemical treatments employ precipitation and ion exchange to modify the chemical properties of the contaminants. Biological methods, such as phytoremediation, utilize metal-accumulating plants to extract and accumulate heavy metals from the soil [110]. The selection of a specific remediation method depends on several factors, including the type and concentration of the heavy metal, prevailing environmental conditions, and the desired level of remediation. Immobilization, soil washing, and phytoremediation techniques are commonly regarded as some of the most effective and well-established technologies for remediating heavy-metal-contaminated sites. Immobilization aims to reduce heavy metal mobility by altering their chemical form, while soil washing involves removing contaminants through the use of chemical or physical agents (Figure 3). Phytoremediation exploits the ability of certain plants to absorb and accumulate heavy metals, effectively mitigating contamination in the soil [111,112,113]. Biochar is a carbon-rich material produced from biomass through a process called pyrolysis. It has gained attention as a potential tool for heavy metal remediation in soil and water due to its unique properties. Biochar can effectively sorb heavy metals, reducing their bioavailability and mobility in the environment [114]. Biochar is a substance that is created through the pyrolysis process of biomass derived from sources such as agriculture, sewage, and animal wastes. Several studies have indicated that biochar has the ability to trap and store pollutants in soil. Additionally, biochar has the capacity to modify the physical and chemical properties of soil in various ways. It can enhance the soil’s ability to retain water, facilitate the uptake of water and minerals by plants, absorb heavy metals, improve soil fertility, and promote a healthy microbiome [92,115,116,117].

The remediation of soil plays a vital role in reducing the risks posed by heavy metal contamination, reclaiming land for agricultural purposes, enhancing food security, and addressing land tenure concerns. Immobilization, soil washing, and phytoremediation are frequently acknowledged as successful approaches for remediating soils contaminated with heavy metals. It is worth noting that these methods have predominantly been demonstrated and employed in developed countries [118].

Recent research has focused on integrated processes to address heavy metal remediation. One such approach is the electrokinetic-geosynthetic method which employs geosynthetic materials and electric current to enhance pollutant mobility, leading to more efficient metal removal from contaminated soil. Another integrated approach involves using permeable reactive barriers combined with microbes, where contaminants are filtered out as water flows through the treated barrier area. These barriers incorporate microbes and/or plants with the capacity to absorb heavy metals from groundwater [119]. Biotechnological methods are also gaining recognition for treating contaminated sediments. Understanding the mechanisms triggered by metal exposure is vital for developing effective remediation strategies, considering the severe health implications associated with heavy metal exposure, including fertility issues and genetic, epigenetic, and biochemical alterations [120]. The complexity and uniqueness of contaminated sites resulting from heavy metals necessitate tailored remediation options. Lastly, strict implementation of regulations by government agencies and decisive actions against industries responsible for toxic environmental discharges are vital for significant reductions in heavy metal levels in the environment [121].

Phytoremediation is a cost-effective and eco-friendly method for tackling soil pollution caused by heavy metals, utilizing plants to remove contaminants. To optimize the effectiveness of phytoremediation, it is essential to develop a deeper comprehension of the mechanisms that govern heavy metal accumulation and tolerance in plants. When plants are exposed to heavy metals, they can generate excessive reactive oxygen species (ROS), leading to oxidative stress. Consequently, boosting antioxidant activity is a commonly employed approach to enhance heavy metal tolerance, fortifying the plant’s defense system against oxidative stress [111,113]. Genetic engineering can be employed to increase heavy metal accumulation in plants. This approach involves introducing and overexpressing genes associated with the uptake, translocation, and sequestration of heavy metals [122]. Another promising strategy is the use of metal chelators, which can be promoted through genetic engineering by overexpressing genes encoding natural chelators. This approach improves heavy metal uptake and translocation in plants [123].

The utilization of plant-associated microorganisms, particularly those found in the rhizosphere, presents another promising strategy for enhancing phytoremediation. The microbial community in the rhizosphere can directly influence plant growth by stimulating root proliferation. This, in turn, leads to improved plant growth, increased tolerance to heavy metals, and overall enhanced plant fitness. By harnessing the beneficial interactions between plants and rhizospheric microorganisms, phytoremediation efforts can be further optimized, providing a more efficient and effective means of addressing heavy metal pollution in soil [113,124,125,126,127].

## 7. Conclusions and Future

ROS produced by heavy metals can harm plants in a variety of ways, including enzyme inhibition, protein oxidation, lipid peroxidation, and DNA and RNA damage. ROS plays a number of important roles in regulating the expression of many genes under normal circumstances. The cell cycle, plant growth, abiotic stress responses, systemic signaling, programmed cell death, pathogen defense, and development are all influenced by reactive oxygen species. The ability of plants to tolerate heavy metals (HMs) is facilitated by their cellular and molecular mechanisms, which allow them to avoid, tolerate, detoxify, and sequester these metals. These mechanisms work together to limit the accumulation of HMs in plant tissues. Metal ion transporters play a crucial role in the uptake, transport, efflux, and sequestration of metal ions, making them a potential target for genetic modification to reduce the absorption and transport of HMs. Recent advancements in biotechnology and genetic engineering have made it possible to develop transgenic plants that can tolerate HM toxicity by employing various types of transporter proteins and transcription factors involved in the uptake, translocation, and compartmentalization of HMs. Heavy metals produce morphological, physiological, and biochemical dysfunctions in plants, which can have a variety of serious consequences. WRKY, ANAC019, ANAC016, and MYB72 transcription factors have been shown to regulate the expression of genes involved in ROS scavenging and heavy metal detoxification. Soil microbes have been found to be beneficial for plants as they can enhance the solubilization of HMs and improve plant growth. Future research should focus on understanding the mechanisms of microbial interaction and HM mobilization in plants, as well as the pathways through which HMs are transported from the roots to the aboveground parts of plants via the xylem. This knowledge will contribute to the development of more effective strategies for managing HM contamination in the environment. Enhancing plant performance is a crucial element in the advancement of efficient phytoremediation methods. Relying on a single approach alone is neither feasible nor satisfactory for the successful remediation of soil polluted with heavy metals. The integration of various strategies, such as genetic engineering, microbe-assisted techniques, and chelate-assisted methods, is imperative to achieve highly efficient and comprehensive phytoremediation.

## Figures and Tables

**Table 1:** Enzymatic and non-enzymatic antioxidants against heavy metal stress.

S. No	Enzymatic Antioxidants	Mode of Action
1.	Superoxide Dismutase (SOD)	SOD is an enzyme that converts superoxide radicals (O^2−^) into hydrogen peroxide (H_2_O_2_) and oxygen (O_2_), preventing the accumulation of harmful superoxide radicals that can damage cells through oxidative stress.
2.	Catalase	Catalase is an enzyme that transforms hydrogen peroxide (H_2_O_2_) into water and oxygen, effectively countering the potential toxicity of excess hydrogen peroxide, particularly in the presence of heavy metals.
3.	Glutathione Peroxidase (GPx)	GPx is an enzyme that employs reduced glutathione (GSH) to convert hydrogen peroxide and lipid hydroperoxides into water and corresponding alcohols. Its vital role lies in safeguarding cells against oxidative damage triggered by heavy metals.
4.	Peroxiredoxins	Peroxiredoxins are enzymes that neutralize peroxides, like hydrogen peroxide, using thiol groups in their active sites. They help detoxify ROS from heavy metal exposure.
5.	Glutathione Reductase (GR)	GR is an enzyme that regulates reduced glutathione (GSH) levels by converting oxidized glutathione (GSSG) to its reduced form. This is crucial for upholding cellular redox equilibrium during heavy metal stress.
6.	NAD(P)H Quinone Oxidoreductase 1 (NQO1)	NQO1 is an enzyme that detoxifies by reducing quinones and electrophilic substances, safeguarding cells from oxidative damage due to heavy metals and pollutants.
7.	Selenium-Containing Enzymes	Selenium is in enzymes like glutathione peroxidases and thioredoxin reductases, crucial for antioxidant defense and redox regulation. They counter heavy-metal-triggered oxidative stress.
8.	Cytochrome P450 Enzymes	Certain cytochrome P450 enzymes metabolize heavy metals, converting them into safer forms. This aids in detoxification and defending against heavy metal stress.
**Non-Enzymatic Antioxidants**		
9.	Glutathione (GSH)	Glutathione, a tripeptide (γ-glutamyl-cysteinyl-glycine), is a key intracellular antioxidant. It helps detoxify heavy metals by binding to them and aiding in their elimination. GSH also supports specific detoxification enzymes as a cofactor.
10.	Ascorbic Acid (Vitamin C)	Vitamin C, a water-soluble antioxidant, neutralizes ROS, shielding cells from heavy-metal-triggered oxidative harm. It also indirectly boosts other antioxidants like GSH and vitamin E.
11.	α-Tocopherol (Vitamin E)	Vitamin E, a lipid-soluble antioxidant, safeguards cell membranes by neutralizing lipid peroxyl radicals. It upholds membrane integrity during heavy metal stress.
12.	Carotenoids	Carotenoids like β-carotene, lutein, and zeaxanthin are plant pigments with antioxidants. They counter ROS and shield cells from oxidative harm due to heavy metals.
13.	Phenolic Compounds	Various phenolic compounds, such as flavonoids and polyphenols, are known for their antioxidant properties. They can scavenge ROS and chelate heavy metals, reducing their toxic effects.
14.	Metal Chelators	Certain non-enzymatic antioxidants can bind to heavy metals, creating stable complexes that decrease reactivity and toxicity. Chelators like EDTA and citric acid, for instance, aid in trapping heavy metals and aiding their removal.
15.	Selenium (Se)	Selenium, an essential trace element, functions as an antioxidant and can counteract heavy metal toxicity. Supplementation with selenium has been found to ease oxidative stress caused by heavy metals.
16.	Melatonin	Melatonin, an indoleamine, functions as a potent antioxidant by scavenging ROS, safeguarding cells from oxidative harm due to heavy metals.
